# Comparative Extraction and Bioactive Potential of the Leaf Extracts of Azadirachta indica for Combatting Postoperative Head and Neck Infections: An In Vitro Study

**DOI:** 10.7759/cureus.51303

**Published:** 2023-12-29

**Authors:** Prateek Veerendrakumar S, Mahathi Neralla, Baskar V, Tharini Satheesh

**Affiliations:** 1 Oral and Maxillofacial Surgery, Saveetha Dental College and Hospitals, Saveetha Institute of Medical and Technical Sciences, Saveetha University, Chennai, IND

**Keywords:** improved quality of life, disease, infected tissue, novel extraction, cytotoxicity, innovative antibacterial extraction methods, antioxidant, azadirachta indica, neem

## Abstract

Introduction: Surgical site infections (SSIs) following head and neck cancer surgery are very common postoperative sequelae. Delayed wound healing leads to a poor aesthetic outcome, delay in restarting oral intake, and delay in getting or starting adjuvant therapy. Antibiotic resistance is on the rise necessitating studies that use alternatives to combat the rising antibiotic resistance. Many plant compounds have been studied to explore the possibility. Neem (*Azadirachta indica*), a high medicinal value plant, possesses a vast array of phytocompounds, which are broadly grouped into isoprenoids and non-isoprenoids. These phytocompounds are crucial for its anti-inflammatory, antioxidant, antimicrobial, antipyretic, and various other pharmacological activities.

Materials and methods: In this study, we examined the impact of the extraction solvents on the bioactive potential of neem. Neem leaf samples were extracted with water and ethanol; followed by their biological activities like extraction yield, antioxidant, antimicrobial, and cytotoxicity studies were performed. The extraction yield was found to be higher in the ethanolic extract than in the aqueous extract, which also corroborates with increased antioxidant and antibacterial activity. Both the aqueous and ethanolic extracts of neem exhibited antibacterial activities against dental biofilm-producing pathogens like *Staphylococcus aureus, Streptococcus mutans, Pseudomonas aeruginosa, and Escherichia coli.*

Results: Extraction yield was higher in the ethanolic extract of neem. Antioxidant activity was found to be higher in the ethanolic extract than in the aqueous extract. Neem extract has no toxicity, which was observed through hemolytic and zebrafish embryo toxicity assays. The ethanolic extract of neem was shown to be more effective against the Gram-positive and Gram-negative drug-resistant bacterial pathogen

Discussion and conclusion: Thus, the utilization of neem extracts is certainly useful in controlling pathogenic bacterial growth in clinical applications. Further, a detailed mechanism of action of neem extract in bacterial growth inhibition at the molecular level is warranted to utilize their potential in disease management.

## Introduction

Cancers of the head and neck including the oral cavity are a major public health concern, with about 650,000 cases in total, and cause 330,000 deaths per year [1]. Head and neck malignancies include cancers of not only the oral cavity but also salivary glands, scalp, ears, eyes, throat, nose, and paranasal sinuses. The location of these malignancies interferes with essential life activities like swallowing and breathing, and others such as the ability to speak, hear, see, smell, and taste [1].

In head and neck malignancies, there is a gender predilection towards men. Excessive alcohol and cigarette intake were identified as key risk factors for more than two decades, which makes them the causative factor of not only squamous cell carcinoma but also lymphoma and sarcoma. As a result, a comprehensive approach is required, including surgery, radiation, chemotherapy, reconstructive surgery, speech therapy, and psychological support. Wide excision and reconstruction are the most often used treatments to enhance cure rates [1]. The postoperative period following surgery should be short to make way for the patient to get adjuvant treatments where necessary.

Despite antibiotic prophylaxis, surgical site infections (SSIs) following head and neck cancer surgery can develop in up to 10% of patients. The CDC defines SSIs as infections occurring within the first 30 days of surgery and involving at least one of several factors, including purulent drainage, positive culture, and either a deliberate incision and drainage or the presence of supporting signs and symptoms [2]. SSIs can cause major consequences such as wound disintegration, mucocutaneous fistula, sepsis, and death. Delayed wound healing leads to a poor aesthetic outcome, delay in restarting oral intake, and delay in getting or starting adjuvant therapy [2].

SSIs can be because of the type, shape and size of the plate material used, or comorbidities of the patient like his conducive medical history. However, when the surgical site becomes infected, the prognosis suffers dramatically, resulting in greater treatment costs, a longer length of time in the hospital, and a delay in other treatments such as chemo or radiation [3]. Antibiotic resistance is also a growing threat globally and alternatives to combat this problem are the need of the hour. This mandates the development of nature-based drug molecules to impede the development of multidrug-resistant microbial species.

Vrana (wound) and its care have been dealt with from the Vedic time to the present day. It has been an important issue since the beginning of medical research. Surgical site wound infection has been a major hindrance in the healing process, and it was expected that with the development of antibiotics, this problem would be solved. Several antibiotics, both systemic and local, have been tried since then, but the problem of persistent wound infection persists. Many therapeutic herbs are mentioned in Ayurveda, the Indian traditional system of medicine, for wound infection. Scientists seeking innovative pharmaceuticals from natural resources are turning to Ayurveda since phytomedicines are not only inexpensive but also effective and comparatively safe [4].

*Azadirachta* *indica *(*A. indica*) is commonly called the neem tree and is grown or native to tropical forests; it is abundantly grown in India. From time immemorial, neem has been valued for its several therapeutic characteristics, including those used in agriculture for non-toxic insect management and traditional medicine for a variety of common human maladies. The neem has antidiabetic, antioxidant, antipyretic, antifungal, antiviral, antiparasitic, antibacterial, contraceptive, antidermatitic, anticancer, and anti-inflammatory properties. Apart from the stem, bark, and roots, even flowers, seeds, and fruits of *A*. *indica *have been utilized as a home remedy for human ailments. It can be a potential source of antimicrobials to be used by the medical fraternity from all branches [5]. 

The objective of this work is to evaluate the influence of extraction methods on the bioactive potential of the medicinally important neem plant so that its effectiveness as an antibiotic can be assessed. To achieve this, neem leaf samples were extracted with water and ethanol and their biological activities like extraction yield, and antioxidant, antimicrobial and cytotoxic properties were assessed.

## Materials and methods

Sample collection and extraction

Leaf samples of *A. indica* were collected and cleaned with distilled water before being ground to a fine powder with liquid nitrogen. One gram of neem powder was blended with 10 mL of distilled water (aqueous) and ethanol before being agitated for two hours at 120 rpm. The extracts were then spun at 2000 rpm in a centrifuge, and the contents were separated via Whatman Grade 1 filter paper (Cytiva, Marlborough, Massachusetts, United States). The filtrates were then collected and kept at -20°C until further use.

Percentage Recovery Yield of Extraction

The yield percentage was computed using the formula: the extraction yield (%) = (weight of extract (g)/weight of plant sample (g)) 100. 

Analysis of DPPH (2,2-Diphenyl-1-Picrylhydrazyl) Activity

The DPPH approach was utilised to determine the neem scavenging potential using the method by Baskar et al [6]. The (%) of scavenging activity = ((Ao - As)/Ao) x 100. Ao is the optical density (OD) value of the control and As is the absorbance of the neem extract.

Evaluation of In Vitro Toxicity

The toxicity of plant extracts in vitro towards zebrafish embryos was investigated. Neem extracts of varying concentrations were tested for mortality in zebrafish over a certain period of time and compared to untreated embryos. The toxicity of neem extract in Hank's solution was tested against 25 selected zebrafish embryos at distinct periods (24, 48, 72, 96, and 120 hours) according to the Organisation for Economic Co-operation and Development (OECD)-203 guidelines [7]. Later, the eggs were relocated to a separate well for the development of the head, tail, and eyes, which were viewed in a microscope (40X). 

Antibacterial Susceptibility Assay

The bacterial susceptibility of the plant extract is tested using the Kirby-Bauer disc diffusion method against human bacterial infection-causing pathogens such as Gram (+)ve (*Staphylococcus aureus (S. aureus), Streptococcus mutans (S. mutans)*), and Gram (-)ve (*Pseudomonas aeruginosa (P. aeruginosa) and Escherichia coli (E. coli)*) bacteria [8]. For the susceptibility test, bacterial cultures were disseminated on Muller-Hinton agar plates. Standard drugs for Gram-positive and Gram-negative bacterial cells included amoxicillin and tetracycline. The plates with 5-mm wells are filled with the matching standard antibiotic, ethanolic neem extract, and aqueous neem extract. All plates were stored for 24 hours at 37°C, and the findings were recorded as inhibitory zones in mm.

Hemolytic Activity Test

The hemolysis test was utilized to assess the hemolytic feature of neem extracts [9]. Ten millilitres of drawn blood was kept in venipuncture tubes with ethylenediaminetetraacetic acid (EDTA), and centrifuged at 1500 rpm (15 minutes at 25 °C) to separate erythrocytes from plasma. 10 mL of phosphate-buffered saline (PBS) (pH 7.4) is used to was it 3 times next. Erythrocytes were added with PBS (5% v/v) for the assays. First, these were protected from light and next the erythrocyte suspensions were mixed in concentrations of 100µg/mL to 1000µg/mL at 37 °C for about 30 minutes with diluted PBS extracts in 2 mL Eppendorf microfuge tubes (Eppendorf SE, Hamburg, Germany). Next, 200µL of supernatant fluids were collected and deposited on a 96-well flat-bottomed microplate after the treatments were spun for three minutes at 13,000 rpm. The hemolysis activity was evaluated by assessing the ODs at 540 nm, and the half-maximal inhibitory concentration (IC50) values were computed as the sample concentration needed to hemolyze 50% of human RBCs. The following formula was used to compute the percentage of hemolysis: hemolysis % = OD treatment - OD negative control/ OD positive control/ - OD negative control x 100

Statistical analysis

Values were denoted as means of triple analyses of the samples (n = 3), plus standard deviation. The data was subsequently evaluated using analysis of variance (ANOVA) and Duncan's multiple range test (p < 0.05) using IBM SPSS Statistics for Windows, Version 21, (Released 2012; IBM Corp., Armonk, New York, United States), a statistical package application.

## Results

Extraction yield

**Figure 1:**
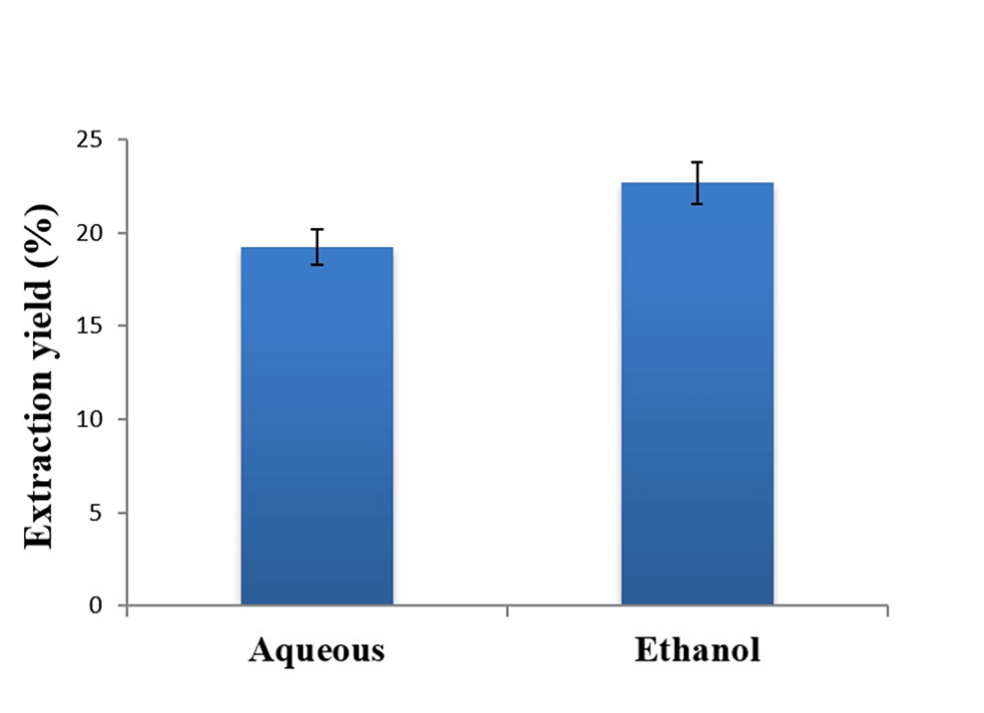
Extraction yields of aqueous and ethanolic extracts

The extraction yield percentages of the aqueous and ethanolic neem leaf extracts were investigated. The extraction yield percentage in the ethanolic extract was found to be greater (22.66%) than in the aqueous extract (19.20%) (Figure 1).

Antioxidant potential of neem extract

**Figure 2:**
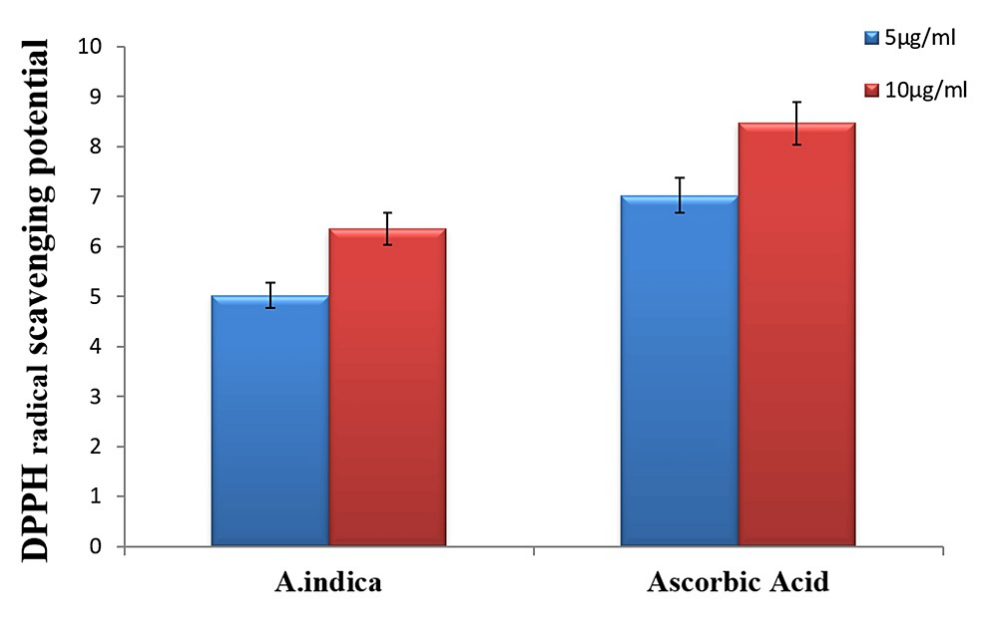
Antioxidant potential of neem extracts

DPPH radical scavenging assay was used to assess the antioxidant effectiveness of several neem extracts. Ascorbic acid was utilised as a control, and the radical scavenging capability of the neem ethanolic extract was equivalent to that of ascorbic acid and higher than that of the aqueous extract. All treatments demonstrated concentration-dependent antioxidant effectiveness (Figure 2). At greater concentrations (10µg/ml), the antioxidant activity increased. 

Antibacterial efficacy of neem extracts

**Figure 3:**
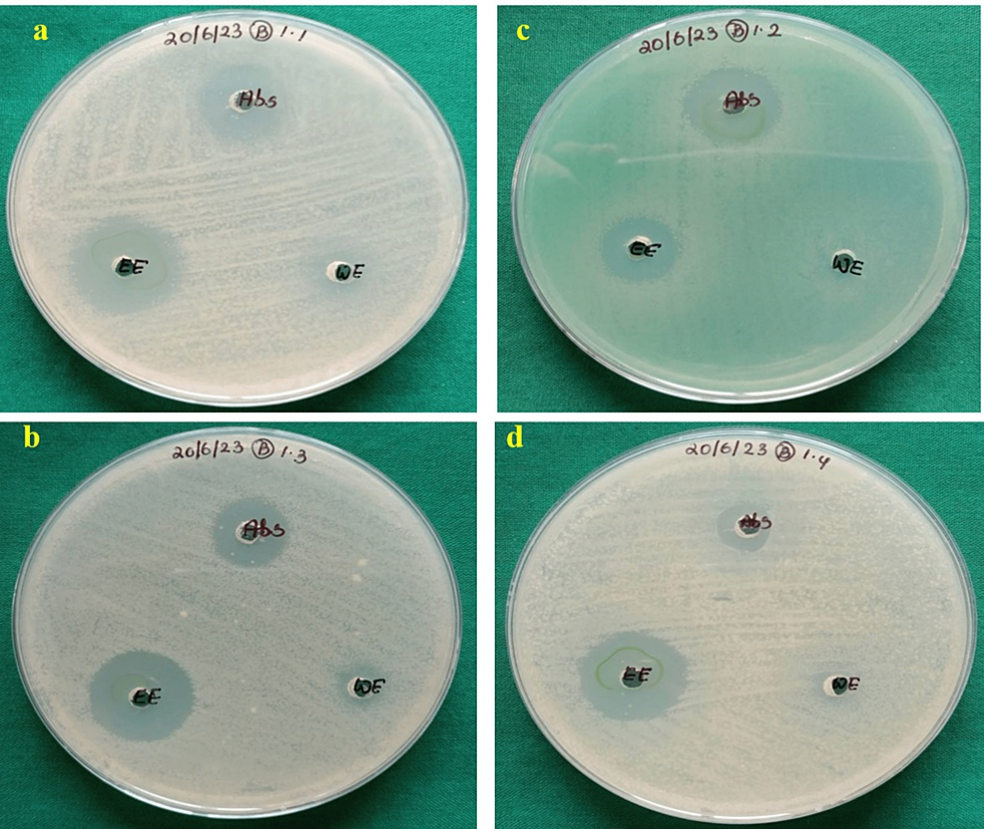
Antibacterial efficacy of neem extracts a-*Staphylococcus aureus*, c-*Streptococcus mutans*, b-*Pseudomonas aeruginosa*, d-*Escherichia coli* ABS: antibiotic; EE: ethanolic extract; WE: water (aqueous) extract

The antibacterial properties of different neem extracts were studied against common nosocomial Gram-positive pathogens, *S. aureus* and *S. mutans*, as well as Gram-negative bacterial species, *P. aeruginosa *and *E. coli.* The ethanolic extracts of neem were more effective against Gram-positive and Gram-negative bacteria than the positive control medication (amoxicillin and tetracycline) and the aqueous extract. *S. mutans* (19 mm) and *E. coli *(16 mm) were more susceptible to ethanolic neem extract (Figure 3 and Table 1).

**Table 1:** Antibacterial efficacy of neem extracts

S.No	Microorganisms (Gram +ve)	Amoxicillin	Aqueous extract	Ethanol extract
		Zone of inhibition in mm		
1	Staphyloccous aureus	14	11	17
2	Streptococcus mutans	13	10	19
S.No	Microorganisms (Gram -ve)	Tetracycline	Aqueous extract	Ethanol extract
1	Pseudomonas aeruginosa	14	10	13
2	Escherichia coli	11	10	16

Impact of ethanolic extract of neem on zebrafish embryos

Ethanolic extract was shown to have a high extraction yield, and antioxidant and antibacterial activity, and its impact on toxicity in biological systems was investigated using a zebrafish embryo toxicity experiment. The harmful impact of an ethanolic extract of neem was examined using a zebrafish embryo toxicity experiment at various time intervals ranging from 24 hours (one day) to 120 hours (five days). There were no clear harmful effects of ethanolic neem extract, which is comparable to the control treated with distilled water. The mortality rate of embryos treated with ethanolic neem extract was somewhat higher depending on the time intervals but was comparable to that of control embryos (Table 2).

**Table 2:** Impact of ethanolic extract of neem on zebrafish embryos Numbers indicate % of dead embryos after treatment with samples. Each value in the table is shown as mean ± SD (n = 3); p < 0.05 - statistically significant.

Time in hours (h)	Control (mortality %)	Sample (mortality %)
24h	0.23	0.34
48h	0.34	0.43
72h	0.42	0.54
96 h	0.65	0.76
120 h	0.98	1.03

Hemolytic activity test

Similarly, the hemolytic activity test was used to investigate the effect of ethanolic neem extract on RBCs. The distilled water had higher hemolytic activity, whereas the ethanolic extract of neem (0.55%) had little hemolytic activity. The results of hemolytic activity showed that neem extract did not exhibit hemolytic activity, implying that neem extract has no deleterious effects and is considered harmless (Table 3).

**Table 3:** Hemolytic activity test of the neem extract OD: Optical density; SE: Standard error

Samples	OD values	SE
Positive control	1.429	1.753
Azadiracta indica	0.047	1.852

## Discussion

The therapeutic potential of various phytochemical substances like *A.* *indica* with their medical uses and biological activity has not been explored much and considerable research is needed in that direction. In Nepal and India, *A*.* indica* is used as a medication for a variety of ailments and infections. The flavonoids and phenolic compounds, apart from tannins, glycosides, and saponins, along with alkaloids, are the phytochemicals found in *A. indica,* which contribute to the antibacterial and antioxidant activities of the leaf extracts [9,10].

Medicinal plants are a reliable and strong source of both traditional and contemporary treatments. With the rising prevalence of multidrug-resistant bacteria, powerful and efficient antimicrobial drugs are required. Plants may be the most abundant source of such bioactive chemicals. Because methanolic extract was employed, this antibacterial action was attributed mostly to polar chemicals found in the leaf extract [8]. Ethanolic extracts had broad-spectrum antimicrobial activity (Gram (+)ve and Gram (-)ve bacteria were susceptible). The antibacterial action towards numerous Gram (+)ve and Gram (-)ve bacteria was attributed mostly to polar chemicals found in the leaf extracts of *Dalbergia* *spinosa* Roxb. [10,11].

An in vitro antioxidant activity test revealed that the plant under investigation contained several potentially antioxidant components. The antioxidant activity was assessed in terms of percentage inhibition of DPPH free radicals, and it was discovered that increasing concentrations of plant extract resulted in higher percentage inhibition. This might be owing to the presence of a high concentration of antioxidant molecules. The presence of flavonoids in plant extracts, as revealed by phytochemical analysis, may be responsible for this antioxidant action [12-14]. The presence of antioxidant and antibacterial activity in the crude leaf extract of *A. indica* validates its therapeutic properties.

In our investigation, ethanol extracts of *A.* *indica* were shown to be more sensitive than standard erythromycin as an antibiotic. *A.* *indica* stem bark extracts also showed considerable antioxidant activity, establishing the extracts' function as antioxidants [13-15]. Similarly, ethanolic extracts of neem leaf showed increased antioxidant activity compared with the aqueous extracts. 

Furthermore, various polarity solvents were used, which most likely eliminated different chemical groups. Ethanol and aqueous extracts revealed the highest radical scavenging activity in both assays, followed by ethyl acetate extract. Previous studies found that polar solvent-extracted neem extracts have stronger antioxidant activity [13]. This study showed a dose-response relationship for DPPH radical scavenging activities; when the dose was increased, the activity increased.

*E.* *coli* and *Salmonella* *typhimurium* are food-borne bacteria, which are susceptible to both aqueous and ethanolic extracts of neem. Amongst the two, ethanolic extracts had the lowest minimum inhibitory concentration (MIC) values compared to the other, even demonstrating good antifungal activity [16-18]. Similarly, the ethanolic and aqueous extracts of neem exhibited potent antibacterial efficacy against drug-resistant bacterial species like *S. aureus, S. mutans*, *E.* *coli,* and *P. aeruginosa,* which are commonly encountered in the postoperative period. In our investigation, the tested bacterial species were shown to be more sensitive in ethanol extracts of *A. indica* than in standard drugs, amoxicillin and tetracycline.

The potential for host cell injury is a crucial pharmacological characteristic of any putative antibacterial agent. To investigate this hypothesis, the neem extract's hemolytic activity was measured after one hour of incubation with RBCs. The findings indicate that neem extract is non-hemolytic at bactericidal doses [19]. In addition, the impact of toxic phenomena of neem leaf extract was examined in vitro in the embryos of zebrafish and the results revealed that there was no obvious toxicity in the embryos, which suggests supportive evidence for the utilization of neem extracts in therapeutic applications [20-21]. 

This is only an in vitro study and detailed molecular studies are warranted to dissect their pharmacological targets and therapeutic potential. This is the main limitation of the present study.

## Conclusions

Drug resistance is a common phenomenon in different types of post-cancer treatment including oral cancer. Natural plant products are viable and sustainable alternatives to synthetic drugs for the treatment of various drug-resistant microbes. Neem is one of the potent antibacterial medicinal plants due to its number of phytomolecules. We compared the extraction yield and bioactive potential of aqueous and ethanolic extracts of neem leaves. Extraction yield and antioxidant activities were higher in the ethanolic extracts, which also correlates with the increased antibacterial activity. Further, no considerable toxic impacts of studied neem leaf extracts were seen in the in vitro toxicity studies. Altogether, our results suggest that the ethanolic extraction of neem leaves is more effective in their utilisation in therapeutic applications. However, detailed molecular studies are warranted to dissect their pharmacological targets and therapeutic potential.
